# Endobronchial cryobiopsy or forceps biopsy for lung cancer diagnosis

**DOI:** 10.4103/1817-1737.69117

**Published:** 2010

**Authors:** Zafer Aktas, Ersin Gunay, Nevin Taci Hoca, Aydin Yilmaz, Funda Demirag, Sibel Gunay, Tugrul Sipit, Emine Bahar Kurt

**Affiliations:** *Department of Chest Diseases, Ataturk Chest Diseases and Thoracic Surgery Training and Research Hospital, Ankara, Turkey*; 1 *Department of Pathology, Ataturk Chest Diseases and Thoracic Surgery Training and Research Hospital, Ankara, Turkey*; 2 *Department of Chest Diseases, Abant Izzet Baysal University School of Medicine, Bolu, Turkey*

**Keywords:** Biopsy, cryobiopsy, cryotherapy, interventional bronchoscopy, lung cancer

## Abstract

**BACKGROUND:**

Invasive procedures such as bronchoscopic biopsy, bronchial washing, and bronchial brushing are widely used in diagnosis of lung cancers. The mean diagnostic rate with bronchoscopic forceps biopsy is 74% in central tumors. This study was designed to evaluate the efficacy of cryobiopsies in histopathological diagnosis.

**METHODS:**

Forty-one patients who had interventional bronchoscopy were included in this study. Three forceps biopsies and one cryobiopsy with cryorecanalization probe were obtained from each subject. Biopsies interpretations were done by one expert pathologist.

**RESULTS:**

Hemorrhage was the only complication in both procedures. There was no significant difference between these two procedures in the incidence of hemorrhage (*P* > 0.05). Mean diameters of samples taken with forceps biopsy and cryoprobe biopsy were 0.2 and 0.8 cm, respectively (*P* < 0.001). Thirty-two patients (78%) were diagnosed with forceps biopsies, and 38 patients (92.7%) were diagnosed with cryoprobe biopsies (*P* = 0.031).

**CONCLUSIONS:**

We concluded that cryoprobe biopsies were more successful than forceps biopsies in diagnosis. Nevertheless, further investigations are warranted to determine an efficacy of cryoprobe biopsy procedures and a rationale to use as a part of routine flexible bronchoscopy.

Lung cancer, the most frequent cause of cancer-related death, is responsible for more than 1 million deaths annually.[1,2] The determination of histopathological cell type and stage of primary lung carcinoma is crucial to develop appropriate treatment approach that affects morbidity and mortality.[3,4] Invasive methods widely using in histopathological diagnosis of lung cancer are bronchoscopic mucosal biopsy, bronchial washing, bronchial brushing, and transthoracic needle aspiration.[4] While the diagnostic rate of bronchoscopic forceps biopsy alone in central tumors is from 65% to 82%, this rate may be increased to around 90% with forceps biopsy combined with bronchial washing and/or bronchial brushing.[5]

Cryotherapy has been used in endobronchial treatments for years.[6] Cryoprobe which was routinely used in cryotherapy is not resistant to tractions so it is not proper for emergent recanalisations. Hetzel and Schumann described and used a novel cryoprobe (ERBE, Medizintechnik GmbH, Tübingen, Germany) which has greater stability to tractions (50 N) and larger surface area than routine cryotherapy probe, in emergent endobronchial recanalisation.[7,8] In cryorecanalisation procedure, in contrast to cryotherapy, after freezing, tumor tissue is pulled with cryorecanalisation probe throughout airways. By this way, larger tissue samples could be taken from endobronchial lesions. Hetzel *et al*.[9] also formerly reported a study regarding a quality of cryobiopsy of exophytic endobronchial lesions in histological diagnosis. They reported that tissue samples obtaining with cryoprobe showed an extraordinary good quality in terms of size and artifact-free sample area. Molecular markers were better preserved as well. Babiak *et al*.[10] also reported the study of transbronchial cryobiopsy for diagnosis of diffuse lung diseases.

To the best knowledge, this is the first study aiming to evaluate the accuracy and efficacy of cryobiopsies in histopathological diagnosis of endobronchial exophytic lesions.

## Methods

The study was performed prospective study in Ataturk Chest Diseases and Thoracic Surgery Training and Research Hospital, Ankara, Turkey between October 2005 and May 2008.

### Characteristic of study group

The patients were recruited from inpatient clinics. Fourty-one patients older than 20 with endobronchial exophytic lesions indicated interventional bronchoscopy were included in this study. The patients who were younger than 20 or had lesions with external compression were excluded.

This study was approved by local ethics committee. Informed consents were obtained from each subject.

### Cryoprobe

We used a flexible cryorecanalization probe (78 cm in length/2.4 mm in diameter) which has higher freezing capacity, higher stability to traction, and larger surface area than conventional cryoprobes for cryotherapy. The instrument in our study (ERBE, Medizintechnik GmbH, Tübingen, Germany) uses nitrous oxide which induces a temperature of -89.5°C at the tip of the probe.

### Application of anesthesia and intubation of patient

After local anesthesia inhalation (2%, prilocain HCl), midazolam (0.05 mg/kg for induction) and propofol (1 mg/kg for induction and 50 μg/kg/min for maintenance) was used for sedation. Patients were intubated with endotracheal tube (Bronchoflex, 7.5 mm; Rüsch GmbH, Kernen, Germany) under 6–12 lt/min oxygen supply.

### Application of bronchoscopy and collection of specimens

Interventional bronchoscopy with fiberoptic bronchoscope was performed by pulmonary diseases specialists. Three forceps biopsies and then one cryoprobe biopsy with flexible cryorecanalization probe were obtained, respectively, while patients were spontaneously breathing. Cryoprobe biopsy was obtained by tumor tissue pulled with cryoprobe throughout airways after freezing (about 20 s) that was occurred at the tip of probe while being embedded into the lesion. Frozen biopsy material was separated from cryoprobe by the way of plunging into saline. Cold water (+4°C saline), diluted adrenalin, and argon plasma coagulator (APC) were provided for possible bleeding complications.

### Evaluation and control of complications

Preoperative and postoperative complications related to anesthesia, intubation, and biopsies were recorded. When hemorrhage could not be controlled following biopsies, interventions including cold water, diluted adrenalin, or APC were applied until hemorrhage being controlled, respectively. Amount of hemorrhage that occurred during biopsy procedures was defined as: mild bleeding (if ice-cold NaCl or adrenalin solution was necessary), moderate bleeding (if argon plasma coagulation was required), and severe bleeding (events with hemodynamic instability).[8]

### Pathological evaluation

After named with different codes, biopsies were evaluated and reported by one pathology specialist based on larger diameter size and histopathological findings. In this examination, first of all, biopsies were fixed separately with 10% buffered formaline filled bottle. Sections were examined under light microscope after stained with hemotoxylin and eosin (H and E). Diagnosis was made according to World Health Organisation (WHO) lung cancer recommendations.[11]

### Statistical analysis

Statistical analysis was done with SPSS 11.5 software program (Statistical Package for Social Sciences, SPSS Inc., Chicago, IL, USA). Distributions of continuous variables were evaluated by Shapiro Wilk test. Descriptive statistics were indicated as follows: mean ± standard deviation for age, median (min.–max.) for pack-year (smoking) and biopsy size, and percentage of nominal variables. Size of biopsies taken via cryoprobe or forceps was analyzed by Wilcoxon-sign test. Diagnostic success rate and incidence of complications were compared with McNemar’s test. Level of agreement among complications following biopsies were assigned based on the value of Kappa coefficient (K). A *P* value less than 0.05 was considered statistical significant.

## Results

Four female (9.8%) and 37 male (90.2%) patients were included in this study with age range from 27 to 75. Mean age was 57.83 ± 10.88. Largest size of the mass on thorax CT of 35 cases was ≥3 cm in length. Endobronchial lesions were localized predominantly in both main bronchi with 34.1% for each. Other characteristics of the patients, CT findings, and bronchoscopical localizations were summarized in Table 1.

**Table 1:** Characteristics of the patients and the lesions (*N*=41)

Characteristics	N	%
Patient population		
Sex		
Male	37	90.2
Female	4	9.8
Age (year) (X ± SD)	57.83 ± 10.88	
Smoking habits		
Smoker	40	97.6
Nonsmoker	1	2.4
Amount of smoking (package-year)[Median (min. – max.)]	40 (0–120)	
CT findings (tumor size)		
≥3 cm	35	85.4
<3 cm	6	14.6
Localization of the lesion		
Right main bronchus	14	34.1
Left main bronchus	14	34.1
Trachea	8	19.5
Right upper lobe orifice	3	7.3
Main carina	2	4.9

Any complication related to anesthesia or intubation could not be determined. Only hemorrhage was seen as a complication in both methods after biopsies. It was found that 34.1% and 36.6% hemorrhage was occurred following forceps and cryoprobe biopsies, respectively. The difference between forceps biopsies and cryoprobe biopsies by meaning of complication was not statistically significant (*P* > 0.05). Level of agreement of complication following both biopsy procedures was found compatible (K: 84.0%, *P* < 0.001). Need of interventions for hemorrhage was summarized in Table 2. APC was applied for hemorrhage (moderate hemorrhage) of two cases following cryoprobe biopsies with the exception of forceps biopsies. Any severe hemorrhage with hemodynamic instability that would need to apply rigid bronchoscopy, surgery, or fluid replacement was not determined during study.

**Table 2:** Types of interventions for hemorrhage following forceps and cryoprobe biopsies (*N*=41)

Types of interventions	Forceps biopsy N (%)	Cryoprobe biopsy N (%)
Simultaneously clotting	5 (12.2)	5 (12.2)
Cold water application	8 (19.5)	5 (12.2)
Adrenalin (diluted) application	1 (2.4)	3 (7.3)
APC application	0	2 (4.9)
Total	14 (34.1)	15 (36.6)

Median size of biopsies with cryoprobe and forceps were 0.8 cm (0.3–4.0 cm) and 0.2 cm (0.1–1.0 cm), respectively (*P* < 0.001).

Thirty-two patients (78%) were diagnosed with forceps biopsies, on the other hand, 38 patients (92.7%) were diagnosed with cryoprobe biopsies. In three of cases, both biopsy methods were non-diagnostic (*P* = 0.031) [Table 3]. Furthermore, histopathological examination of tissue samples obtained via cryobiopsy was demonstrated good quality in terms of necrosis and artifact-free sample area.

**Table 3:** Diagnostic rates for forceps and cryoprobe biopsies (*N*=41)

Forceps biopsy	Cryoprobe biopsy		Total, N (%)
	Diagnostic, N (%)	Nondiagnostic, N (%)	
Diagnostic	32 (78)	0	32 (78)
Non-diagnostic	6 (14.6)	3 (7.3)	9 (22)
Total	38 (92.7)	3 (7.3)	41 (100)

Six cases (14.6%) that were nondiagnostic with forceps biopsy were diagnosed with cryoprobe biopsies. Furthermore, 14 cases (34.1%) which were diagnosed as nonsmall cell carcinoma with forceps biopsies were elaborated with cryoprobe biopsies [Table 4].

**Table 4:** Results of histopathological diagnosis with forceps and cryoprobe biopsies (*N*=41)

	Diagnosis with forceps biopsy						
Diagnosis with cryoprobe biopsy	ND	NSCLC	Squamous cell carcinoma	Adeno carcinoma	SCLC	Clear cell carcinoma	Total
ND	3 (7.3)	0	0	0	0	0	3 (7.3)
NSCLC	2 (4.9)	4 (9.8)	0	0	0	0	6 (14.6)
Squamous cell carcinoma	2 (4.9)	9 (22)	14 (34.1)	0	0	0	25 (61)
Adeno carcinoma	1 (2.4)	1 (2.4)	0	1 (2.4)	0	0	3 (7.3)
SCLC	0	0	0	0	2 (4.9)	0	2 (4.9)
Clear cell carcinoma	0	0	0	0	0	1 (2.4)	1 (2.4)
Adenoid cystic carcinoma	1 (2.4)	0	0	0	0	0	1 (2.4)
Total	9 (22)	14 (34.1)	14 (34.1)	1 (2.4)	2 (4.9)	1 (2.4)	41 (100)

SCLC = Small cell lung cancer; NSCLC = Nonsmall cell lung cancer; ND = Nondiagnostic

All the endobronchial lesions located at main carina and right upper lobe were successfully diagnosed by both biopsy modalities. Seven of 8 lesions located at trachea, 8 of 14 at right main bronchi, and 12 of 14 at left main bronchi were diagnosed by forceps biopsies. All lesions located at trachea, 12 of 14 at right main bronchi, and 13 of 14 left main bronchi were diagnosed by cryobiopsy successfully.

## Discussion

Lung cancer is the most prevalent malignant tumor in the world. It was reported that one-third of death related to cancer was result of lung cancer in America and Europe for a year.[12] In spite of all treatments including surgery, about 16% of lung cancer cases could survive for 5 years.[13]

Most of the cases with nonsmall cell lung cancer without any metastasis could be recovered by surgical treatments. Accordingly, determination of histopathological cell type and stage of primary lung carcinoma is crucial in immediate planning of appropriate treatment modality and prognosis.[3,4]

Invasive modalities widely used in diagnosis of lung cancer are bronchoscopic mucosal biopsy, bronchial washing, bronchial brushing, and transthoracic needle aspiration.[4] Factors that effect the success of diagnostic modality are diameter and localization of the mass, and visibility at endobronchial tree with bronchoscope. The most prevalent diagnostic tool in central and exophytic endobronchial lesion is bronchoscopy. Therefore, we performed our biopsy with flexible bronchoscope. Generally, bronchoscopy is a confident diagnostic tool. Complications were rarely reported in the course of procedures. These complications are including vasovagal reactions, postbronchoscopic fever, cardiac arrhythmia, hemorrhage, bronchospasm, pneumonia, pneumothorax, and death. Death during bronchoscopy are reported very rarely.[14] Credle *et al*.[15] reported three deaths in 24,521 cases of bronchoscopy (mortality rate of 0.01%). Hemorrhage related to bronchoscopy is a more common complication. Severity of bleeding is related to biopsy type. More common and more severe hemorrhage was reported with transbronchial biopsies when compared with endobronchial biopsies. Rate of hemorrhage was reported at 1–26%.[14] In our study, after biopsies were obtained, only hemorrhage was seen as a complication in both methods. The difference between forceps biopsies and cryoprobe biopsies by meaning of hemorrhage was not statistically significant (*P* > 0.05). Death or other complications were not reported in our series.

Cordasco *et al*.[14] reported that susceptibility to hemorrhage was related to bleeding diathesis, thrombocytopenia, defect in thrombocytic functions [secondary to medical treatments or diseases (e.g. uremia)], immune deficiency (drug induced), lymphoma, leukemia, bronchogenic carcinoma, and HIV. In this study, all the patients were immunocompetent and there was no coagulopathy, thrombocytopenia, and uremia. However, all patients had been diagnosed carcinoma (bronchogenic carcinoma and lung metastasis of genitourinery truct carcinoma).

Bronchoscopic methods widely used in diagnosis of lung cancer are forceps biopsy, bronchial washing, and bronchial brushing. Among these methods, bronchial washing alone was reported as the lowest success rate in diagnosis of central lesions (48%).[4] Otherwise, diagnostic rate of bronchial brushing alone in central tumors was about 50%.[16] Only one diagnostic procedure, especially the cytology alone, may cause some mistakes in diagnosis. Most widely preferred invasive diagnostic method in central exophytic lesions is bronchoscopic forceps biopsy. Success rate diminishes when the lesion becomes more distal. The mean diagnostic rate of bronchoscopic forceps biopsy in central tumors is 74%.[4] In our study, diagnostic rate of forceps biopsy was 78% and diagnostic rate of cryobiopsy was 92.7% (*P* = 0.031).

Combined methods of forceps biopsy, bronchial washing, and brushing, altogether, may improve the success of diagnosis up to 97.3%. Therefore, combination at least two methods had been suggested in the literature.[4,17] We used cryobiopsy alone as a diagnostic tool. Further investigations are warranted to evaluate the combined methods with washing and/or brushing.

Importance of number of biopsies was studied in literature as well. Multiple bronchoscopical biopsies are also better for diagnosis of lung cancer. It was advised that at least three mucosal biopsies must be taken before determination of bronchoscopy for enough sampling.[4,5] We also performed sample collection with three mucosal biopsies with forceps biopsy; however, only one biopsy was taken for each cryobiopsy. However, only one cryobiopsy was found more successful than the three forceps biopsies (*P* = 0.031).

In the case of accuracy of diagnosis, it is known that size of the specimen was important. It was reported that the largest sample that can be taken via forceps biopsies was 0.5 cm in diameter.[18] In our study, while the median size of the biopsies taken via forceps was 0.2 cm (0.1–1.0 cm) in diameter, the median size of the cryobiopsies was 0.8 cm (0.3–4 cm). There was a significant difference between the sizes of biopsy specimen (*P* < 0.001). In the recent studies, tissue samples with cryoprobe were found good quality in biopsy-size without morphological artifact.[8,19] We also considered that one of the main reasons that provide higher diagnostic success for cryobiopsy was the larger biopsy specimen.

In our study, diagnosis of lung metastasis of clear cell carcinoma was achieved by both procedures. However, diagnosis of adenoid cystic carcinoma was only obtained by cryobiopsy. It was reported that diagnosis of adenoid cystic carcinoma (cylindroma) which is rare neoplasm arising from salivary, lacrimal, or other exocrine glands can be obtained by forceps biopsies, fine-needle aspiration cytology.[20,21] However, in the case of nondiagnostic forceps biopsies for the less common tumors, before using more invasive diagnostic operations such as mediastinoscopy or thoracotomy it should be considered more confident to take biopsies with cryobiopsy.

Differential diagnosis of NSCLC and SCLC can be made with biopsy specimens; even it is so small, without any discrepancy in pathologist.[22] It was reported that adequacy and size of the specimens may improve the histological accuracy of NSCLC.[1,10] In this study, 14 patients were diagnosed with NSCLC by forceps biopsies. On the other hand, 9 of these 14 patients were diagnosed with epidermoid carcinoma; only one patient was diagnosed as adenocarcinoma. Only four patients were diagnosed similarly with the forceps and cryoprobe biopsies. These results demonstrate that cryobiopsies are predominant in the case of detailing NSCLC in comparison with forceps biopsies.

To our knowledge, this is the first study that compare diagnostic efficacy of cryobiopsy and forceps biopsy in the case of exophytic endobronchial lesions. In this study, we found that cryobiopsy has more successful diagnostic results than forceps biopsies. Complications (only hemorrhage) during procedures were similar in both biopsies. The most important factor to obtain more diagnostic biopsies via cryoprobe are the size of the biopsies and good quality in terms of artifact-free sample area. As a result, we consider that cryobiopsy is a successful and confident diagnostic method that can be used during fiberoptic broncoscopy under local anesthesia. Nevertheless, further investigations are warranted to determine efficacy of cryoprobe biopsy procedures and a rationale to use as a part of routine bronchoscopy and further studies are needed to compare diagnostical efficacy of cryoprobe biopsy and larger cup forceps biopsy via rigid bronchoscopy.
